# Weight Loss Induced by Bariatric Surgery Restricts Hepatic *GDF15* Expression

**DOI:** 10.1155/2018/7108075

**Published:** 2018-11-08

**Authors:** Timon E. Adolph, Felix Grabherr, Lisa Mayr, Christoph Grander, Barbara Enrich, Alexander R. Moschen, Herbert Tilg

**Affiliations:** Department of Internal Medicine I, Gastroenterology, Hepatology, Endocrinology & Metabolism, Medical University Innsbruck, Innsbruck 6020, Austria

## Abstract

**Introduction:**

Obesity and related nonalcoholic fatty liver disease (NAFLD) are an emerging health care issue that imposes substantial morbidity to individuals. Growth and differentiation factor 15 (*GDF15*) limits food uptake, body weight, and energy balance by modulation of GDNF-family receptor *α*-like (GFRAL) signalling in the hindbrain. However, the regulation of *GDF15* expression in obesity and NAFLD is incompletely understood. We sought to define the impact of weight loss achieved by laparoscopic adjustable gastric banding (LAGB) on hepatic and adipose *GDF15* expression in a cohort of severely obese patients.

**Methods:**

We analysed *GDF15* expression of liver and subcutaneous adipose tissue before and 6 months after LAGB in severely obese patients undergoing LAGB by quantitative real-time PCR. To assess the role of inflammation on *GDF15* expression, we analysed Hep G2 hepatocytes stimulated with cytokines such as IL-1*β*, TNF*α*, IL-6, LPS, or cellular stressors such as tunicamycin.

**Results:**

*GDF15* expression was mostly confined to the liver compared to adipose tissue in severely obese patients. Weight loss induced by LAGB was associated with reduced hepatic (but not adipose tissue) expression of *GDF15*. Stimulation with IL-1*β* or tunicamycin induced hepatic *GDF15* expression in hepatocytes. In line with this, hepatic *GDF15* expression directly correlated with IL-1*β* expression and steatosis severity in NAFLD. These data demonstrated that amelioration of metabolic inflammation and weight loss reduced hepatic *GDF15* expression.

**Conclusion:**

Based on recent mechanistic findings, our data suggest that hepatic *GDF15* may serve as a negative feedback mechanism to control energy balance in NAFLD.

## 1. Introduction

Nonalcoholic fatty liver disease (NAFLD) and obesity are dramatically increasing worldwide. Systemic inflammation and tissue inflammation represent a critical driver of disease processes in obesity and its related disorders including NAFLD [1, 2]. We previously described that hepatic inflammation in NAFLD patients could be reversed by weight loss that was achieved by laparoscopic adjustable gastric banding (LAGB). LAGB not only improved metabolic dysregulation but also liver disease [3, 4].

Growth and differentiation factor 15 (*GDF15*), also known as MIC-1, is a member of the transforming growth factor *β* (TGF-*β*) family. Increased tumor-derived *GDF15* concentrations mediated cachexia by modulation of food intake in mice [5]. In line with these findings, overexpression of *GDF15* in mice reduced food intake and body weight while genetic deletion of *GDF15* evoked obesity [6, 7], and administration of recombinant *GDF15* ameliorated diet-induced obesity in mice [8]. Only recently, a mechanism of *GDF15*-controlled food intake and body mass has been revealed in a series of reports in *Nature Medicine* [9–11]. These studies demonstrated that GDNF-family receptor *α*-like (GFRAL) served as a receptor of *GDF15* signalling in the hindbrain (i.e., area postrema and nucleus tractus solitarius) which was required for the metabolic effects of *GDF15* [9–11]. Specifically, mice exposed to a high-fat diet exhibited decreased food intake and body weight when they were treated with recombinant *GDF15* which was likely mediated by signalling in the brain [9]. In line with this notion, intracerebroventricular *GDF15* application in rats similarly resulted in reduced food intake [11]. Knockout models established that GFRAL signalling particularly protected against diet-induced obesity, while no phenotype was observed at baseline [9–11]. These data suggest that *GDF15*/GFRAL signalling critically controls energy balance in a situation with unrestricted dietary access to high-caloric food. In line with this, *GDF15*-mediated GFRAL signalling at the brainstem regulated food intake and energy expenditure in metabolic and toxic-induced stress [12]. As such, clear evidence accumulated that *GDF15* allows limitation of food intake to control body weight under calorie-rich dietary conditions.

Based on these data, *GDF15*/GFRAL signalling emerges as a promising target to treat obesity in the future. However, the regulation of *GDF15* in obesity-related human disease processes is poorly understood. *GDF15* is expressed in the liver [13], and patients with nonalcoholic steatohepatitis exhibited increased systemic *GDF15* level when compared to healthy controls or patients without simple steatosis [14]. In this cohort of NAFLD patients, systemic *GDF15* concentrations increased with hepatic fibrosis and correlated with liver stiffness measured by elastography [14]. Although *GDF15* emerges as a critical driver of metabolism in diet-induced obesity, the impact of body weight on hepatic *GDF15* expression remains unexplored. We tracked a cohort of 28 severely obese patients that underwent laparoscopic adjustable gastric banding (LAGB) and analysed the impact of weight loss on *GDF15* expression in the liver and subcutaneous adipose tissue. We found that *GDF15* expression was mostly confined to the liver and that weight loss induced by LAGB was associated with reduced hepatic (but not adipose tissue) expression of *GDF15*. Mediators of metabolic inflammation such as IL-1*β* and tunicamycin induced hepatic *GDF15* expression in hepatocytes and IL-1*β* expression correlated with *GDF15* expression in the liver of NAFLD patients. As such, weight loss and reduction in low-grade inflammation induced by LAGB in severely obese patients impact on hepatic *GDF15* expression [3, 15]. In light of recent studies [9–12], our findings suggest that hepatic *GDF15* may serve as a feedback mechanism to control energy balance in NAFLD.

## 2. Material and Methods

### 2.1. Study Design

Evaluation for LAGB was performed at the Department of Medicine, Innsbruck Medical University, Innsbruck, Austria. In this study, twenty-eight patients (21 females, 7 male) with a BMI of more than 35 kg/m^2^ were included between 2003 and 2007 [4]. Patients with alcohol intake of more than 20 g per week, statin treatment, or other cause of chronic liver diseases (autoimmune or viral hepatitis, PBC, PSC, haemochromatosis, and Wilson's disease) were excluded from the study. The protocol was approved by the ethics committee of the Medical University Innsbruck, and patients provided written informed consent before LAGB and sample collection. Liver and abdominal subcutaneous tissue specimens were taken intraoperatively at LAGB and per biopsy six months after LAGB along with blood samples from the fasting state. Clinical parameters were assessed, and blood and biopsy specimen were stored at −80°C. Patient characteristics are summarized in Table 1.

### 2.2. Quantification of Hepatic and Adipose mRNA Expression

Expression analysis was performed as previously reported [4]. Tissue samples were thawed and total RNA was extracted using TRIzol® Reagent (Invitrogen, Carlsbad, California). RNA was reverse transcribed using Moloney murine leukemia virus (M-MLV) reverse transcriptase (Invitrogen, Carlsbad, California). Quantitative real-time PCRs were performed with mesa green master mix (Eurogentec, Seraign, Belgium) on an Mx3000 qPCR Cycler (Stratagene, La Jolla, California). Expression was normalised to the housekeeping gene *glyceraldehyde-3-phosphate dehydrogenase* (*GAPDH*). The following primer sequences were used: *GAPDH*, forward: GTC GCC AGC CGA GCC; *GAPDH* reverse: CCC AAT ACG ACC AAA TCC GT; *GDF15*, forward: GAC CCT CAG AGT TGC ACT CC; and *GDF15*, reverse: GCC TGG TTA GCA GGT CCT C.

### 2.3. Culture and Stimulation of Hep G2 Hepatocytes

Hep G2 human hepatocellular carcinoma cells were purchased from ATCC (HB-8065; Middlesex, UK) and cultured in DMEM supplemented with 10% fetal bovine serum and penicillin/streptomycin. Cells were stimulated with lipopolysaccharide (LPS 100 ng/ml; Invivogen, San Diego, California), recombinant human TNF*α* (50 ng/ml; Peprotech), rec. IL-1*β* (1 ng/ml, Peprotech, 200-01B), rec. IL-6 (10 ng/ml, Peprotech, 200-06), or tunicamycin (1 *µ*g/ml, Sigma, T7765) for 24–48 hours overnight.

### 2.4. Histological Analysis of Hepatic Biopsies

Hepatic biopsies were formalin-fixed and paraffin-embedded and stained with hematoxylin and eosin. A blinded pathologist scored the severity of steatosis (0–4) as previously described [15].

### 2.5. Statistical Analysis

Results are expressed mean ± standard error of the mean (SEM) or dot blot where appropriate. Statistical significance between two groups was determined by a two-tailed Student's *t*-test, a Wilcoxon signed-rank test, or a two-way ANOVA where appropriate and considered significant at *P* < 0.05. Linear regression was analysed by GraphPad Prism version 6.0.

## 3. Results

### 3.1. LAGB Ameliorates Metabolic Inflammation

We hypothesised that weight loss induced by laparoscopic gastric banding impacted on *GDF15* expression. We analysed hepatic and subcutaneous fat expression before and 6 months after laparoscopic gastric banding in severely obese patients in a longitudinal fashion [4]. Our cohort comprised 28 patients with an average age of 38 years who had lost 21.9 kg ± 9.76 kg 6 months after LAGB (Table 1) [4]. Weight loss was paralleled by reduced low grade systemic inflammation indicated by leukocyte counts and C-reactive protein (CRP). Furthermore, weight loss was associated with reduced hepatic injury indicated by a reduction in alanine aminotransferase (ALT) and gamma-glutamyltransferase (GGT). In line with this, metabolic inflammation improved after 6 months as demonstrated by an improved homeostasis model assessment (HOMA) index and reduced hepatic expression of inflammatory cytokines such as IL-1*β* and IL-6 (Table 1 and [3, 16, 17]).

### 3.2. LAGB-Induced Weight Loss in Obese Patients is Associated with Reduced Hepatic *GDF15* Expression

**Figure 1:**
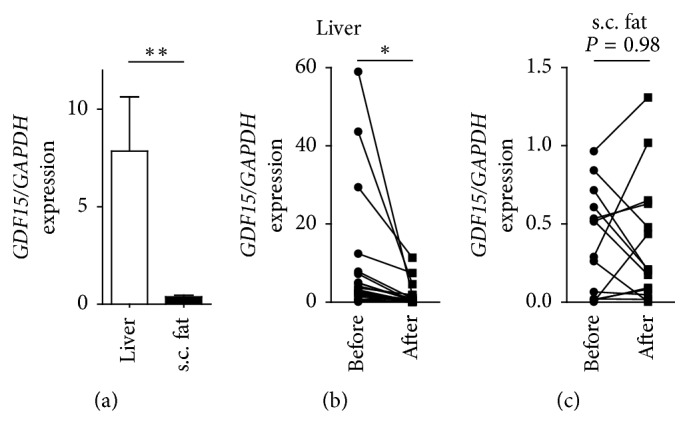
*GDF15* is strongly expressed in the liver of obese subjects and decreases after laparoscopic adjustable gastric banding. (a) Hepatic and subcutaneous adipose tissue *GDF15* expression in obese patients determined by qPCR and normalised to *GAPDH*. (b, c) Hepatic (b) and subcutaneous adipose tissue (c) *GDF15* expression in obese patients before and 6 months after LAGB determined by qPCR and normalised to *GAPDH*. ^*∗*^ *P* < 0.05, ^*∗∗*^ *P* < 0.01.

We utilised this cohort to analyse the expression of *GDF15* in liver and subcutaneous adipose tissue specimens before and 6 months after LAGB. Hepatic *GDF15* expression was largely confined to the liver in obese patients before LAGB (Figure 1(a)). Six months after LAGB, hepatic *GDF15* expression substantially decreased in all individuals while we observed no demonstrable effect in subcutaneous adipose tissue (Figures 1(b) and 1(c)).

### 3.3. Inflammation and Endoplasmic Reticulum Stress Induce *GDF15* Expression

**Figure 2:**
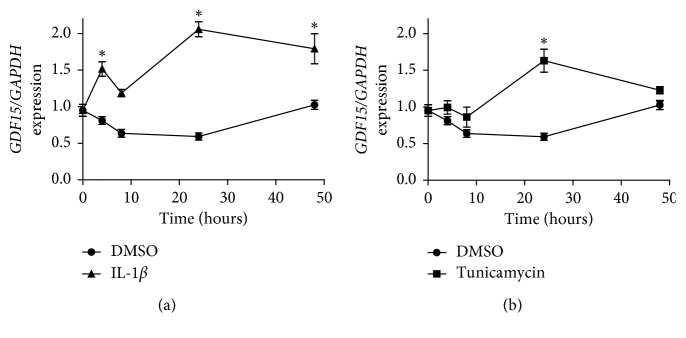
IL-1*β* and tunicamycin promote *GDF15* expression in hepatocytes. (a, b) *GDF15* expression in Hep G2 hepatocytes over the course of 48 hours stimulation with interleukin 1b (a) or the endoplasmic reticulum stressor tunicamycin (b) determined by qPCR and normalised to *GAPDH*. Data from 3 independent experiments are shown. ^*∗*^ *P* < 0.05.

To understand the effect of weight loss on hepatic *GDF15* expression, we utilised Hep G2 hepatocytes as a model system. As hepatic inflammation ameliorated 6 months after LAGB [3, 16, 17], we hypothesised that cytokines and cellular stress may induce the expression of *GDF15*. To address the impact of cytokines and cellular stress on *GDF15* expression, we stimulated Hep G2 cells with IL1-*β*, TNF*α*, IL-6, LPS, and tunicamycin, the latter being an inducer of endoplasmic reticulum stress [18]. We noted that IL-1*β*, but not TNF*α*, IL-6 or LPS, induced the expression of *GDF15* in hepatocytes (Figure 2(a), Supplementary Figure 1). Furthermore, endoplasmic reticulum stress induced by tunicamycin increased the expression of *GDF15* in Hep G2 hepatocytes (Figure 2(b)). These data indicated that hepatic inflammation contributed to increased *GDF15* expression in obese patients [14] which could be reversed by LAGB-induced weight loss.

### 3.4. *GDF15* Expression Correlates with Hepatic Steatosis and IL-1*β* Expression in NAFLD

**Figure 3:**
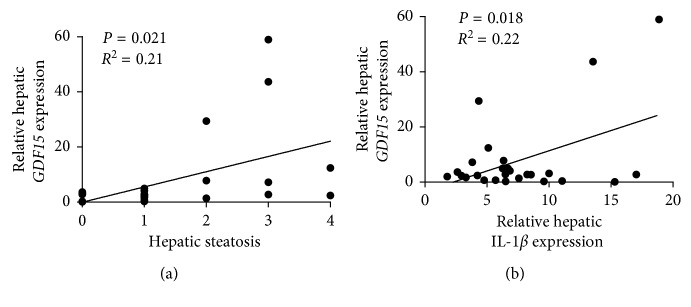
Correlation of hepatic *GDF15* expression with steatosis and inflammation. (a, b) Hepatic *GDF15* mRNA expressions correlated with histologically quantified steatosis (a) and IL-1*β* expression (b). Respective *R* values and level of significance are shown in each panel. Each dot represents individual patient before or after LAGB.

To assess a relationship between the regulation of *GDF15* and metabolic inflammation in NAFLD, we correlated clinical parameters with *GDF15* expression before LAGB. We did not note a correlation between hepatic *GDF15* expression and BMI, HOMA, liver injury, systemic inflammation (C-reactive protein), or hepatic TNFα expression (Supplementary Figures 2(A)–2(E)). In contrast, we noted a direct correlation between hepatic *GDF15* expression and steatosis assessed by histologic means (Figure 3(a)). Furthermore, hepatic expression of *GDF15* correlated with IL-1*β* (Figure 3(b)). These data indicated a direct relationship between features of NAFLD and hepatic *GDF15* expression.

## 4. Discussion

*GDF15* limits food uptake and obesity in experimental models. However, the regulation in and impact on obesity and related diseases in humans are incompletely understood. A previous study demonstrated increased circulating *GDF15* concentrations in advanced NAFLD [14]. We report that hepatic (but not adipose tissue) *GDF15* expression decreased after LAGB-induced weight loss. In hepatocytes, *GDF15* expression was promoted by IL-1*β* signalling and ER stress both of which have been implicated in the development of NAFLD [19, 20]. A previous study demonstrated that palmitic acid impacted on *GDF15* expression particularly in Kupffer cells [14]. Collectively, these findings may explain why LAGB-induced weight loss was associated with reduced hepatic *GDF15* expression as we previously noted reduced hepatic inflammation (i.e., IL-1*β* expression) and improved metabolic dysfunction consequent to bariatric surgery in this cohort [3,15–17].

Previous studies convincingly demonstrated that *GDF15* shapes the susceptibility to developing obesity and that *GDF15* treatment ameliorated diet-induced obesity [8–12]. These data provide the basis for a model in which hepatic *GDF15* is strongly expressed in NAFLD [14] to limit food intake and diet-induced obesity. In line with this, a recent study demonstrated increased hepatic *GDF15* expression in NASH animal models and humans which may protect against NAFLD [21]. *GDF15* signalling may act locally (e.g., in the liver) or systemically which we cannot address in this study. Specifically, we were unable to provide systemic *GDF15* level in our cohort due to lack of sample availability, and *GFRAL* was neither expressed in the liver (as previously demonstrated [10]) nor in adipose tissue of our cohort (data not shown). After LAGB-induced weight loss, which is in part mediated by restricted food uptake [22], a compensatory expression of *GDF15* in the liver may be less pronounced. In line this with notion, *GDF15* expression directly correlated with steatosis severity in our study, a critical feature of NAFLD which could be reverted by weight loss [17]. As such, *GDF15* treatment may be beneficial in obese patients and after LAGB as many patients relapse [22]. In this context, a local inflammatory milieu (e.g., hepatic MIC-1 expression) may also impact on the regulation of body weight [5].

To further explore a therapeutic benefit of *GDF15* in metabolic diseases and NAFLD, additional experimental studies are needed. It may be plausible that *GDF15* mediated actions other than regulation of food intake control susceptibility to obesity and related disorders. For example, *GDF15* may act anti-inflammatory by limiting neutrophilic inflammation as seen in myocardial infarction [23]. This observation appears important in NAFLD, as advanced stages are characterised by hepatic neutrophilic inflammation [24]. Moreover, *GDF15* controls hepatic hepcidin expression and iron overload which may set the susceptibility to NAFLD [25, 26]. Interestingly, *GDF15* serum level is a predictor of all-cause mortality which highlights the importance of *GDF15* signalling in many disease processes [27].

In conclusion, our study demonstrated that weight loss induced by LAGB reduced hepatic *GDF15* expression in patients with NAFLD which may be mediated by a reduction in low-grade inflammation [16]. Based on previous findings [6,9–12], these observations suggest that *GDF15* expression in NAFLD [14] occurs in a compensatory manner and that targeting this pathway may ameliorate obesity and related disorders.

## Figures and Tables

**Table 1:** Clinical characteristics of patients before and after LAGB.

	Before LAGB	After LAGB	*P* value
*N* (female/male)	28 (21/7)	—	—
Age	38 [19–66]	—	—
BMI (kg/m^2^)	43.01 ± 3.70	35.7 ± 4.53	*P* < 0.001
Weight loss (kg)	21.90 ± 9.76	—	—
% excessive weight loss	39.57 ± 17.92	—	—
Fasting glucose (mg/dl)	103.02±17.81	89.47 ± 9.17	*P* < 0.001
Insulin (U/I)	20.85 ± 15.06	11.89 ± 7.87	*P* > 0.001
HOMA	5.53 ± 4.54	2.71 ± 2.05	*P* < 0.001
AST (U/L)	30.59 ± 12.93	25.44 ± 7.14	*P*=0.058
ALT (U/L)	36.45 ± 27.90	23.89 ± 12.30	*P* < 0.05
GGT (U/L)	36.04 ± 24.57	25.64 ± 16.41	*P* < 0.01
AP (U/L)	66.86 ± 17.87	66.00 ± 11.37	*P*=0.838
CRP (mg/dl)	1.01 ± 0.73	0.63 ± 0.35	*P* < 0.05
Leukocyte count (G/L)	7.32 ± 1.88	6.48 ± 1.39	*P* < 0.05

ALT, alanine aminotransferase; AST, aspartate aminotransferase; BMI, body mass index; CRP, C-reactive protein; HOMA, homeostasis model assessment (calculated as Insulin (*µ*U/ml) × glucose (mmol/l)/22.5); GGT, *γ*-glutamyl transferase.

## Data Availability

The data used to support the findings of this study are included within the article.

## Abbreviations

ALT: Alanine aminotransferase
BMI: Body mass index
*GDF15*: Growth differentiation factor 15
GGT: c-Glutamyl transferase
HOMA: Homeostasis model assessment
LAGB: Laparoscopic gastric banding
NAFLD: Nonalcoholic fatty liver disease.

## Additional Points

(i) *GDF15* is strongly expressed in the liver compared to adipose tissue in obesity. (ii) Weight loss induced by LAGB is associated with reduced hepatic *GDF15* expression in obese patients. (iii) Inflammatory signals such as IL-1*β* or unresolved endoplasmic reticulum stress induce *GDF15* expression in hepatocytes. (iv) Hepatic *GDF15* expression directly correlates with features of human NAFLD i.e., IL-1*β* expression and steatosis.

## Supplementary Materials

Supplementary Materials Supplementary Figure 1: IL-6, TNF, and LPS do not impact on *GDF15* expression in hepatocytes. *GDF15* expression in Hep G2 hepatocytes over the course of 24 hours stimulation with IL-6, tumor necrosis factor *α* (TNF*α*), or lipopolysaccharide (LPS) determined by qPCR and normalised to GAPDH. Data from 3 independent experiments are shown. Supplementary Figure 2: correlation of hepatic *GDF15* expression with clinical features. (A–E) Hepatic *GDF15* mRNA expressions did not correlate with body mass index (BMI) (A), homeostasis model assessment (HOMA) index (B), liver injury (C), systemic inflammation (D), and hepatic TNF*α* expression (E). Respective *R* values and level of significance are shown in each panel. Each dot represents an individual patient before or after LAGB.
