# Supplementary figures and images for: Weight Loss Induced by Bariatric Surgery Restricts Hepatic GDF15 Expression
Source: J Obes. 2018 Nov 8;2018:7108075. doi: 10.1155/2018/7108075 (PMC6250003; doi:10.1155/2018/7108075)

Supplementary Figure 1

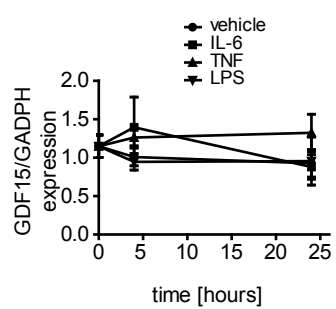

Supplementary Figure 2

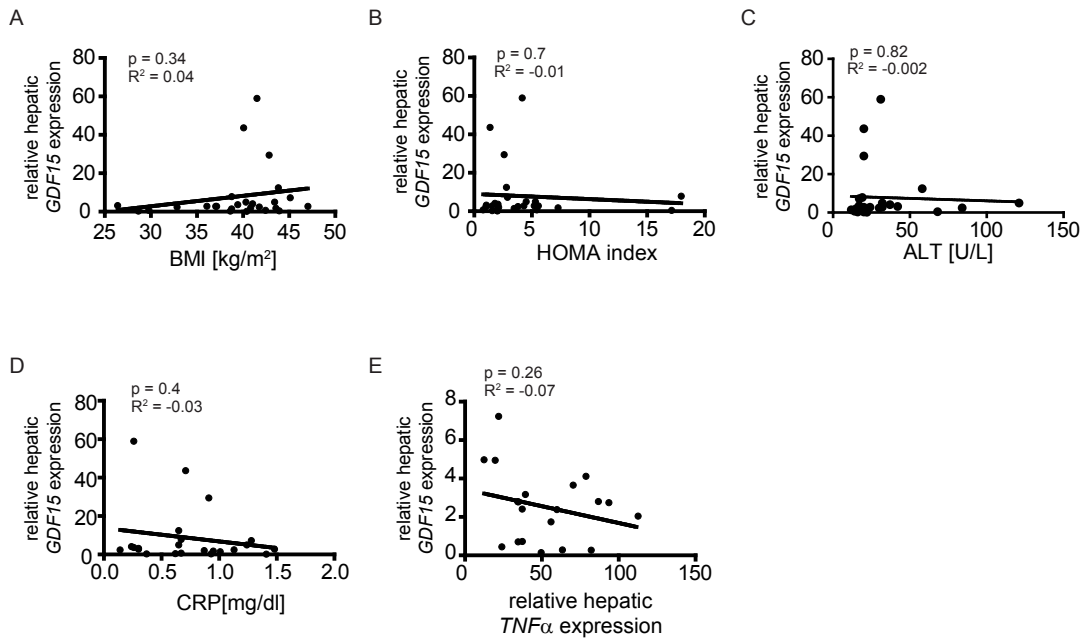

Supplement: Supplementary Materials — Supplementary Figure 1: IL-6, TNF, and LPS do not impact on GDF15 expression in hepatocytes. GDF15 expression in Hep G2 hepatocytes over the course of 24 hours stimulation with IL-6, tumor necrosis factor α (TNFα), or lipopolysaccharide (LPS) determined by qPCR and normalised to GAPDH. Data from 3 independent experiments are shown. Supplementary Figure 2: correlation of hepatic GDF15 expression with clinical features. (A–E) Hepatic GDF15 mRNA expressions did not correlate with body mass index (BMI) (A), homeostasis model assessment (HOMA) index (B), liver injury (C), systemic inflammation (D), and hepatic TNFα expression (E). Respective R values and level of significance are shown in each panel. Each dot represents an individual patient before or after LAGB. [file 7108075.f1.pdf]
